# Slow flow induces endothelial dysfunction by regulating thioredoxin-interacting protein-mediated oxidative metabolism and vascular inflammation

**DOI:** 10.3389/fcvm.2022.1064375

**Published:** 2022-11-16

**Authors:** Yongshun Wang, Jingjin Liu, Huadong Liu, Xin Sun, Ruimian Chen, Bihong Liao, Xiaoyi Zeng, Xiaoxin Zhang, Shaohong Dong, Zhengyuan Xia, Jie Yuan

**Affiliations:** ^1^Department of Cardiology, Shenzhen People’s Hospital, The First Affiliated Hospital of Southern University of Science and Technology, The Second Clinical Medical College, Jinan University, Shenzhen, China; ^2^State Key Laboratory of Pharmaceutical Biotechnology, Department of Medicine, The University of Hong Kong, Hong Kong, Hong Kong SAR, China; ^3^Department of Anesthesiology, Affiliated Hospital of Guangdong Medical University, Zhanjiang, China

**Keywords:** disturbed flow, thioredoxin-interacting protein (TXNIP), endothelial dysfunction, mitochondrial dysfunction, oxidative metabolism

## Abstract

Endothelial cells are highly sensitive to hemodynamic shear stresses, which act in the blood flow’s direction on the blood vessel’s luminal surface. Thus, endothelial cells on that surface are exposed to various physiological and pathological stimuli, such as disturbed flow-induced shear stress, which may exert effects on adaptive vascular diameter or structural wall remodeling. Here we showed that plasma thioredoxin-interactive protein (TXNIP) and malondialdehyde levels were significantly increased in patients with slow coronary flow. In addition, human endothelial cells exposed to disturbed flow exhibited increased levels of TXNIP *in vitro*. On the other hand, deletion of human endothelial TXNIP increased capillary formation, nitric oxide production and mitochondrial function, as well as lessened oxidative stress response and endothelial cell inflammation. Additional beneficial impacts from TXNIP deletion were also seen in a glucose utilization study, as reflected by augmented glucose uptake, lactate secretion and extracellular acidification rate. Taken together, our results suggested that TXNIP is a key component involved in mediating shear stress-induced inflammation, energy homeostasis, and glucose utilization, and that TXNIP may serve as a potentially novel endothelial dysfunction regulator.

## Introduction

The coronary slow flow phenomenon (CSFP) is an important, angiographic entity characterized by delayed progression of the injected contrast medium, in the absence of significant epicardial coronary stenosis (1). Coronary angiograms in patients with CSFP are often referred to as “normal” or “mild non-obstructive disease.” Although it is well-known to interventional cardiologists for approximately four decades, the pathogenic mechanisms are incompletely understood. The mechanism of CSFP may involve endothelial function, inflammatory, and oxidative stress processes.

Shear stress is one of primary mechanical forces normally experienced by large arteries (2). Mechanically stimulated release of potent shear-responsive factors from endothelial cells regulates vessel tone and structure (3–5). This process is facilitated by the endothelium being sensitive to hemodynamic shear stresses acting on the vessel luminal surface in the direction of blood flow. By extension, physiological and pathological variations of shear stress, caused by multiple pathophysiological conditions such as hyperlipidemia, hypertension, diabetes and inflammatory disorders, regulate endothelium-dependent changes in vascular diameter in an acute manner, and sustained shear stress induce slowly adaptive structural wall remodeling (6).

Shear stress spans a range of spatiotemporal scales and give rise to regional and focal heterogeneity of endothelial gene expression. This process is important in the evolution of vascular pathology. Thioredoxin-interactive protein (TXNIP) is known to promote oxidative stress by binding and subsequently inhibiting thioredoxin activity (7, 8). In doing so, TXNIP is reported to influence cardiac metabolism, including mitochondrial function and glucose uptake (9). Researchers have also demonstrated that TXNIP modulates cellular glucose utilization and mitochondrial oxidation of metabolic fuels (5, 10–12). On the other hand, TXNIP-null mice cannot survive prolonged fasting, exhibiting dysglycemia and dyslipidemia (11). Besides its involvement in cellular redox and energy metabolism, increasing evidence points toward TXNIP having an important role in vascular function and inflammation process. Studies in endothelial cells showed that TXNIP promotes inflammatory response in response to disturbed flow (13) and arterial stiffness (14, 15). This pro-inflammatory effect is confirmed by the finding that it is required for NLRP3 inflammasome activation and IL-1β production in cultured THP-1 cells (16).

However, the role of TXNIP in endothelial dysfunction is not well addressed. Given the important role of TXNIP in redox homeostasis and inflammation, we hypothesized that its ablation would protect endothelial cells from oxidative stress-induced damage and reduce vascular inflammation. In the present study, we tried to explain the mechanism of microvascular dysfunction by exploring endothelial function, inflammatory, and oxidative stress. We used a disturbed-flow model to investigate the effects of TXNIP deletion on cellular redox status and inflammatory response. Our data demonstrated that TXNIP plays an important role in the development of endothelial dysfunction.

## Materials and methods

### Study population

Patients were enrolled with coronary slow-flow phenomenon (*n* = 16), defined *via* coronary angiography based on either a reduced Thrombolysis in Myocardial Infarction (TIMI) flow grade of 2, were randomly recruited at the Department of Cardiology, Shenzhen People’s Hospital. Exclusion criteria were: Coronary stenosis of more than 50%, Second/third-degree atrioventricular block, ventricular arrhythmia history, myocardial infarction or coronary revascularization, uncontrolled HF, significant renal/hepatic disease, severe COPD or aortic stenosis, acute pulmonary embolism/myocarditis, symptomatic cerebrovascular disease within 12 months, or expected mortality in ≤ 12 months. Control individuals were enrolled with atherosclerosis of coronary (*n* = 20) defined *via* coronary angiography and age- and sex-matched with coronary slow flow patients. In brief, in patients of slow flow, we collected 10 ml of blood from the coronary or peripheral artery from the same patients. To account for dilutions, all analyses were normalized to hematocrit. All patients provided written informed consent. The study was approved by the Committee for Medical and Health Ethics of Shenzhen People’s Hospital, Jinan University.

### Cell culture and disturbed flow treatment

Human umbilical vein endothelial cells (HUVECs) were purchased from the American Type Culture Collection (Manassas, VA, USA), and were grown under culturing condition with Dulbecco’s modified Eagle’s medium containing 10% fetal bovine serum (FBS), 50 U/ml penicillin and 50 μg/ml streptomycin (Invitrogen, Carlsbad, CA, USA), as specified by the manufacturer. To initiate disturbed flow treatment, confluent HUVECs, seeded onto collagen I-coated glass slides, were assembled into flow chambers and connected to the flow system for the shear experiments. HUVECs were exposed to steady laminar flow shear stress (12 dyn/cm^2^), disturbed flow shear stress (0.5 ± 4 dyn/cm^2^) for 24 h.

### Small interfering RNA transfection

Small interfering RNAs (SiRNAs) were used to silence the TXNIP expression. The TXNIP-siRNA duplex was synthesized by Shanghai GenePharma Co., Ltd., (sense: 5′CUCCCUGCUAUAUGGAUGUTT-3′; anti-sense: 5′-ACAUCCAUAUAGCAGGGAGTT-3′). The cells, treated with either the transfection reagents (vehicle) or non-targeting siRNA (sense: 5′-UUCUCCGAACGUGUCACGUTT-3′; anti-sense: 5′-ACGUGACACGUUC GGAGGAGAATT-3′), served as controls. The cells were transfected with 200 nM siRNA using the X-treme siRNA Transfection Reagent (Roche Applied Science, Penzberg, Germany), following the manufacturer’s instructions. Three experimental groups were conducted: the treatment group of laminar flow with negative control siRNA (LF + NC-siRNA), disturbed flow with negative control siRNA (DF + NC-siRNA) and disturbed flow with TXNIP-siRNA (DF + TXNIP-siRNA).

### Plasma TXNIP and MDA measurements by using ELISA

Blood samples were collected from patients and centrifuged at 3000 rpm for 10 min at 4°C. Plasma TXNIP concentration was measured using human TXNIP and malondialdehyde ELISA kit (MyBioSource), according to the manufacturer’s instructions.

### Western Blot analysis

Protein samples with equal amount of total protein (20 μg) were separated on SDS-PAGE (8–15%). The separated protein gel was then transferred to polyvinylidene difluoride membranes (Millipore, Billerica, MA, USA). After blocking with 5% non-fat milk at room temperature for 1 h in Tris-buffered saline containing 0.1% Tween-20, primary antibody incubation (TXNIP, Cat#: 14715; Thioredoxin 1, Cat#: 2285; GLUT4, Cat#: 2213; pyruvate dehydrogenase E1-alpha [PDH E1α], Cat#: 31866; p-eNOS, Cat#: 9571; Total-eNOS, Cat#: 5880; NLRP3, Cat#: 13158; VCAM-1, Cat#: 13662; ICAM-1, Cat#: 4915; Cleaved-IL-1β, Cat#: 83186 and GAPDH, Cat#: 5174 were all from Cell Signaling Technology; Anti-Nitro tyrosine antibody [Cat#: ab42789, Abcam Company]) was carried out overnight at 4°C. Afterward, secondary antibody incubation with a peroxidase-conjugated AffiniPure goat anti-rabbit or anti-mouse IgG was conducted for 90 min at room temperature. After washing 3 times, the membranes were subjected to ECL detection. Densitometric analysis was performed using the Tanon Gel Imaging System (Shanghai Tanon, Shanghai, China). The housekeeping gene GAPDH served as a loading control.

### Tube formation assay

Two hundred μl of Biocoat Matrigel (Becton Dickinson) was added into each well in the 24-well plate and incubated at 37°C for 30 min to solidify. The same batch of Matrigel was used for all the experiments. After flow velocity treatment, 5*10^6^ cells were suspended in culture medium and plated on the Matrigel-coated plate. Gels were examined using a phase-contrast microscope equipped with a digital camera at 72 h after plating. Capillary-like structures were assessed and quantified by calculating the number of junctions per field. At least 5 different viewing fields per well were analyzed.

### Measurement of nitric oxide production

The generation of intracellular nitric oxide (NO) was monitored using the 4-amino-5-methylamino-2,7’-difluorofluorescein (DAF-FM DA) reagent (Beyotime Institute of Biotechnology). HUVECs were incubated with DAF-FM DA solution at 37°C for 30 min. After washing cells three times with PBS, fluorescent intensity was determined at an excitation wavelength of 488 nm and an emission wavelength of 525 nm *via* a fluorescent microplate reader (SpectraMax M2, Molecular Devices Corp., USA).

### Mitochondrial isolation and measurement of ATP levels

For mitochondrial isolation, HUVECs were manually homogenized using a medium-fitting glass Teflon Potter-Elvehjem homogenizer in isolation buffer (mitochondrial isolation buffer: 250 mM sucrose, 0.5 mM EDTA, 10 mM Tris, and 0.1% BSA at pH 7.4). The homogenate was then clarified through centrifuging two times at 1000 × *g* for 5 min, followed by centrifugation twice more at 11000 × *g* for 10 min. The resulting supernatant and mitochondrial pellets were collected and diluted with mitochondrial isolation buffer three times of the original volume.

Mitochondrial ATP was measured by the mitochondrial ToxGlo assay according to the manufacturer’s protocol. Briefly, isolated HUVECs mitochondria were plated at 1 mg/well in both white and clear bottomed 96-well culture plates. The assay solution (100 μL/well) was then added, and the plate was incubated at room temperature for 30 min. Luminescence was measured using a luminometer (Molecular Devices).

### Mitochondrial reactive oxygen species levels

Isolated mitochondria were doubly stained with MitoTracker Red (0.5 μM; excitation/emission 550/590 nm, Invitrogen Company) and dichlorodihydrofluorescein (DCF) diacetate (10 μM; excitation/emission 488/535 nm, Invitrogen Company). The superoxide levels were examined according to the change in MitoSOX Red fluorescence using a confocal microscopy (Zeiss LSM 780). Mean values were analyzed by CellQuest (ver. 5.2; DB CellQuest Pro).

### Mitochondria membrane potential

Isolated mitochondria were stained for 30 min with 0.1 mM tetramethylrhodamine ethyl ester (Invitrogen Company excitation/emission 564/580 nm) at room temperature and measured by flow cytometry to detect the mitochondrial membrane potential.

### Glucose consumption and lactate secretion

Human umbilical vein endothelial cells were seeded into culture plates and incubated for 5 h. The culture medium was then changed and cells were cultured for another 16 h. The levels of glucose in the culture medium were measured using an assay kit from Nanjing Jiancheng Bioengineering Institute (Nanjing, China), following the manufacturer’s recommendations. Lactate concentration was measured with a Lactate Assay Kit (Biovision Inc.), in accordance with the manufacturer’s instructions. The glucose consumption and lactate secretion were normalized to the cell number.

### Electron microscopy

To determine the mitochondrial morphology, including number, size, and shape, HUVECs were sliced and fixed in 2.5% glutaraldehyde in PBS at 4°C overnight, then fixed under 1% osmium tetroxide in PBS for 2 h. Mitochondrial morphology was observed using an electron microscope, and the number of mitochondria was calculated using ImageJ software.

### Assays for glucose metabolism

Oxygen consumption rate (OCR) and extracellular acidification rate (ECAR) of HUVECs were measured using the Seahorse XF Glycolysis Stress Test Kit on an XF24 Extracellular Flux Analyzer (Agilent Technologies), following the manufacturer’s instructions. Cells were grown under standard growth conditions for 1 day prior to the metabolic analysis.

### Statistical analysis

GraphPad Prism 6.0 software is used to perform statistical analyses. Data are presented as mean ± SD. All pairs were compared to each other *via* either Student’s *t*-test or least significant difference (LSD) test, as appropriate. Experimental mice groups were subject to correlation analyses through Bonferroni’s *post hoc* test. One-way analysis of variance (ANOVA) followed by Tukey’s *post hoc* test for multiple comparisons, were utilized for comparing multiple groups among each other. *P*-values < 0.05 were considered significant.

## Results

### TXNIP were accumulated in the patients of coronary slow flow

**FIGURE 1:**
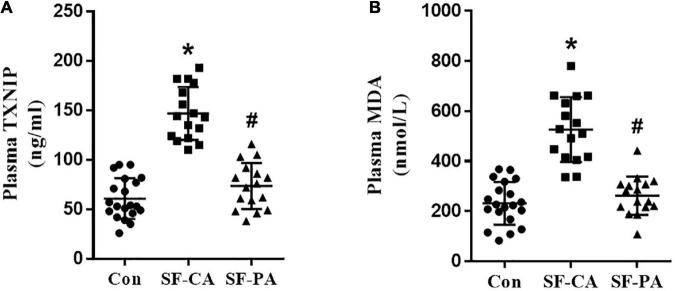
Plasma TXNIP and MDA were elevated in the coronary of patients with coronary slow flow phenomenon. **(A)** Plasma TXNIP was measured by using ELISA; **(B)** Plasma MDA was measured by using ELISA; *P < 0.05 vs. Con, ^#^*P* < 0.05 vs. SF-CA.

To test our hypothesis that slow flow enhance oxidative metabolism, we collected plasma samples from coronary or peripheral artery in coronary slow flow patients or control individuals. Control individuals are age- and sex-matched with patients. Patients with coronary slow flow do not exhibit slow flow in peripheral artery. The baseline characteristics of the human subjects from whom plasma are depicted in Table 1. The plasma TXNIP levels were significantly increased in coronary of slow flow patients compared with control individuals or femoral artery from the same patients (Figure 1A). In addition, the malondialdehyde (MDA), known as a marker of oxidative stress, was significantly elevated in coronary of slow flow patients (Figure 1B). All these findings thus indicate that TXNIP may involve in oxidative disorder in slow flow patient’s coronary.

**TABLE 1:** Demographic and clinical characteristics of study subjects.

Variable	Control	Slow flow	*P*-value
Age (year)	60.9 ± 2.5	60.6 ± 3.0	0.921
Sex (female)	43.75%	45.00%	0.942
SBP (mmHg)	133.9 ± 3.7	135.7 ± 4.0	0.748
DBP (mmHg)	79.00 ± 1.8	81.63 ± 3.4	0.469
HbA1c (%)	5.9 ± 0.23	6.0 ± 0.25	0.612
TG (mmol/L)	4.84 ± 0.11	4.36 ± 0.16	0.018
TC (mmol/L)	2.09 ± 0.16	1.71 ± 0.18	0.120
LDL (mmol/L)	3.30 ± 0.13	2.570 ± 0.16	0.001
HDL (mmol/L)	1.10 ± 0.03	1.22 ± 0.08	0.094
Smoke (%)	56.25%	55.00%	0.942

SBP, systolic blood pressure; DBP, diastolic blood pressure; HbA1c, hemoglobin A1c; TC, total cholesterol; TG, triglyceride; LDL-C, low-density lipoprotein cholesterol; HDL-C, high-density lipoprotein cholesterol.

### Increased expression of TXNIP in disturbed flow induced endothelial dysfunction

**FIGURE 2:**
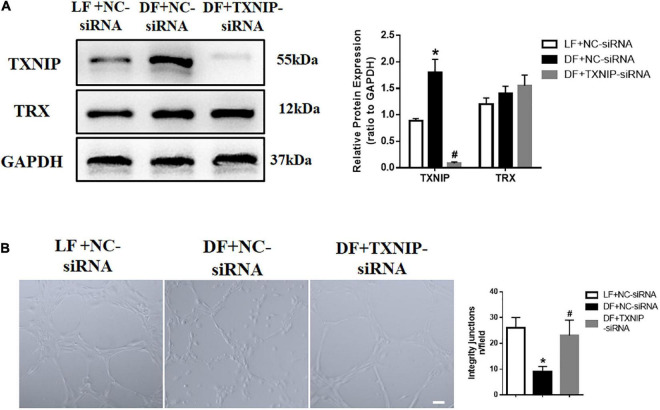
Increased expression of TXNIP in disturbed flow-induced endothelial dysfunction. **(A)** Thioredoxin-interactive protein (TXNIP) expression was determined by Western Blotting in the treatment groups of laminar flow with negative control siRNA (LF + NC-siRNA), disturbed flow with negative control siRNA (DF + NC-siRNA) and disturbed flow with TXNIP-siRNA (DF + TXNIP-siRNA). **(B)** Formation of capillaries in Matrigel. Scale bars = 50 μm. Results are shown as mean ± SD. *N* = 5/group. *P < 0.05 vs. LF + NC-siRNA, ^#^*P* < 0.05 vs. DF + NC-siRNA.

To assess the functional role of flow disturbances on TXNIP expression, the cultured HUVECs were exposed to disturbed flow shear stress (0.4 dyn/cm^2^) or steady laminar flow (12 dyn/cm^2^) for 24 h. TXNIP expression levels in HUVECs were then examined by Western Blotting, which demonstrated that disturbed flow significantly enhanced TXNIP protein levels compared to laminar flow (Figure 2A). The results also showed that the levels of Thioredoxin (TRX), referred to as a small redox protein acting as an electron donor to peroxidases and ribonucleotide reductase, had no changes under disturbed conditions compared to laminar flow. In addition, to investigate whether flow velocity affects endothelial dysfunction, we tested the ability of HUVECs to form capillary-like structures. HUVECs were seeded in Matrigel after disturbed flow or laminar flow treatment, and tube formation was examined microscopically. Further confirmation of disturbed flow inducing endothelial dysfunction through regulating TXNIP expression was queried through treating the cells with either TXNIP-siRNA or negative control siRNA (NC-siRNA). The results showed that the tube structures formed more slowly under disturbed flow conditions, whereas cells subjected to laminar flow exhibited a greater extent of capillary formation (Figure 2B). However, TXNIP-siRNA treatment abrogated the formation of the disconnected capillary-like structures but increased the development of the proper capillary network under disturbed flow conditions (Figure 2B). Collectively, these data suggest that disturbed flow led to a significant TXNIP expression increase, resulting in endothelial dysfunction.

### Disturbed flow reduced production of nitric oxide in a TXNIP dependent manner

**FIGURE 3:**
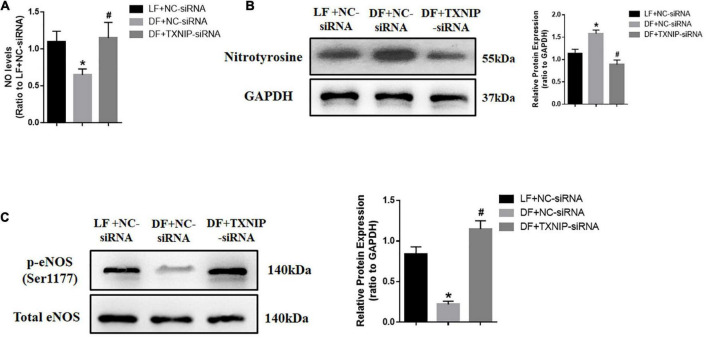
Disturbed flow reduced NO production in a TXNIP dependent manner. **(A)** NO production was assessed with DAF-FM DA, **(B)** Western Blot analysis of nitrotyrosine modified protein expression, **(C)** Western Blots comparing the presence of phosphorylated eNOS at Ser1177 and total eNOS, in the LF + NC-siRNA, DF + NC-siRNA, and DF + TXNIP-siRNA treated groups. Results are shown as mean ± SD. *N* = 5/group. *P < 0.05 vs. LF + NC-siRNA, ^#^*P* < 0.05 vs. DF + NC-siRNA. DAF-FM DA: 4-amino-5-methylamino-2,7’-difluorofluorescein.

Both acute and chronic attenuation in NO production are major factors favoring endothelial dysfunction. To explore the possibility of disturbed flow regulating NO production, we measured NO and nitrotyrosine levels in endothelial cells. The results showed that disturbed flow significantly reduced NO levels and increased nitrotyrosine in HUVECs. By contrast, TXNIP-siRNA treatment attenuated all of these alterations in the DF group (Figures 3A,B). We also measured the protein levels of endothelial nitric oxide synthase (eNOS), which is primarily responsible for vascular endothelial NO generation. Western Blot analysis showed that phosphorylation of eNOS at Ser1177 was downregulated in the disturbed flow with NC-siRNA group, compared with those in steady laminar flow group (Figure 3C). In comparison, the protein level of eNOS at Ser1177 in the DF + TXNIP-siRNA group was upregulated compared to the DF + NC-siRNA group. These results collectively suggested that elevated TXNIP during disturbed flow contributes to eNOS depression and activity.

### Disturbed flow induced endothelial mitochondrial dysfunction through regulating TXNIP expression

**FIGURE 4:**
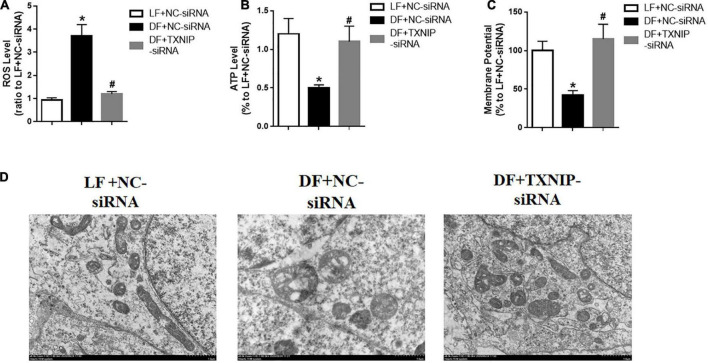
Disturbed flow induced endothelial mitochondrial dysfunction by regulating TXNIP expression. **(A)** Reactive oxygen species (ROS) level, **(B)** Mitochondrial ATP level, **(C)** Mitochondrial membrane potential, in the LF + NC-siRNA, DF + NC-siRNA, and DF + TXNIP-siRNA treated groups. **(D)** Representative image of mitochondrial morphologies with transmission electron microscopy of HUVECs, respectively. Results are shown as mean ± SD. *N* = 5/group. *P < 0.05 vs. LF + NC-siRNA, ^#^*P* < 0.05 vs. DF + NC-siRNA.

Endothelial dysfunction is thought to be mediated mostly by reactive oxygen species (ROS). Mitochondria are the major cellular ROS producers, due to their crucial role in energy metabolism. To explore the possibility of disturbed flow inducing mitochondrial dysfunction, we detected mitochondrial ROS in isolated mitochondria from HUVECs, with or without TXNIP expression. The results showed that disturbed flow treatment exhibited higher levels of ROS compared to laminar flow, while TXNIP-siRNA significantly reduced mitochondrial ROS levels compared to the DF + NC-siRNA group (Figure 4A). Similarly, disturbed flow also reduced ATP levels compared to laminar flow (Figure 4B). By contrast, TXNIP-siRNA treatment in the disturbed flow group resulted in significantly increased ATP levels compared to the DF + NC-siRNA group (Figure 4B). We also observed a remarkable reduction in mitochondrial membrane potential among the disturbed flow group, compared to mitochondria isolated from laminar flow-treated cells. Conversely, TXNIP-siRNA abrogated the reduction found in the disturbed flow group compared to the DF + NC-siRNA group (Figure 4C). Mitochondrial morphology under electron microscopy in disturbed flow-treated HUVECs showed bizarre shapes and poorly defined cristae (Figure 4D). Conversely, TXNIP expression inhibition led to less disorganized mitochondrial morphology compared with that of the DF + NC-siRNA group. These results therefore demonstrated that disturbed flow may aggravate mitochondrial dysfunction by distorting mitochondrial morphological features and increasing ROS levels while silencing TXNIP partially reversed the pathological response in HUVECs.

### Disturbed flow decreased glucose utilization by TXNIP-dependent activation

**FIGURE 5:**
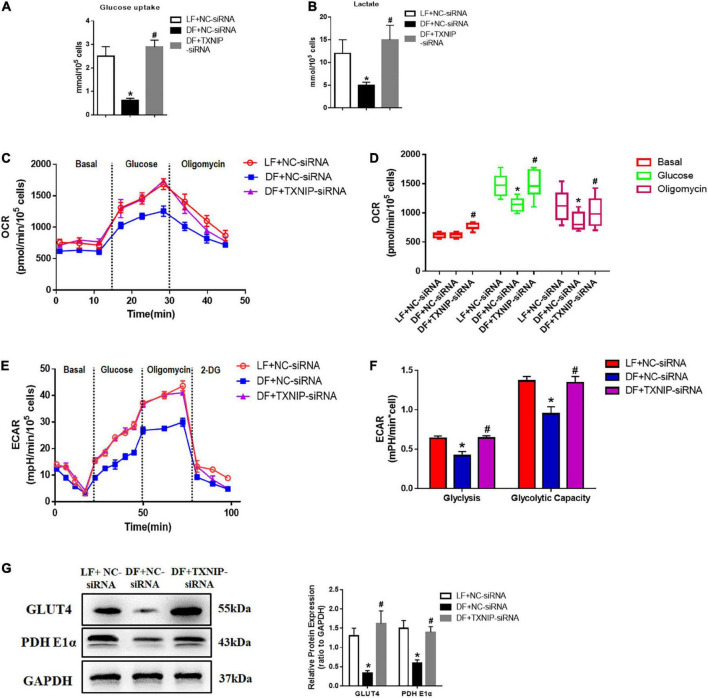
Disturbed flow decreased glucose utilization through TXNIP-dependent activation. **(A)** Glucose uptake, **(B)** Lactate production, **(C)** Kinetic oxygen consumption rate (OCR) responses of HUVECs to 20 mM glucose and 5 mM oligomycin, **(D)** Calculated glucose oxidation rate, **(E)** Kinetic extracellular acidification rate (ECAR) responses of HUVECs to glucose (20 mM), oligomycin (5 μM) and 2-DG (100 mM), in the LF + NC-siRNA, DF + NC-siRNA, and DF + TXNIP-siRNA treatment groups. **(F)** Calculated glycolytic flux and glycolytic capacity. The glycolytic flux and glycolytic capacity are calculated by ECAR increase normalized with cell protein content. **(G)** GLUT4 and PDH E1α expression were determined by Western Blotting in the treatment groups of laminar flow with negative control siRNA (LF + NC-siRNA), disturbed flow with negative control siRNA (DF + NC-siRNA), and disturbed flow with TXNIP-siRNA (DF + TXNIP-siRNA). All values are presented as mean ± SD. *N* = 5/group. *P < 0.05 vs. LF + NC-siRNA, ^#^*P* < 0.05 vs. DF + NC-siRNA.

As TXNIP has been identified as a key determinant of glucose utilization in cardiac metabolism, we investigated glucose uptake and lactate production in disturbed flow-treated HUVECs, with or without TXNIP. Glucose uptake and lactate production in disturbed flow-treated HUVECs was reduced to varying degrees compared to the laminar flow group (Figures 5A,B). It is worth noting that TXNIP depletion completely abrogated the disturbed flow-induced reduction of those aforementioned metabolites compared to the DF + NC-siRNA treatment group (Figures 5A,B). We further examined the impacts of TXNIP deficiency on aerobic metabolism within laminar or disturbed flow groups by measuring the OCR using the XF24 extracellular flux analyze, indicating aerobic metabolism of glucose *via* tricarboxylic acid (TCA) cycle and mitochondrial oxidative phosphorylation. As illustrated in Figures 5C,D glucose or oligomycin (an ATP synthase inhibitor) addition triggered significant OCR increase in the laminar flow group, but only a moderate augmentation in the disturbed flow group. Conversely, TXNIP deletion triggered significant OCR increase in the disturbed flow group compared to the disturbed flow with NC-siRNA (DF + NC-siRNA) group. In addition, to determine the effects of different flow velocities on HUVEC glycolytic flux, varying levels of flow velocity were applied to cells, with or without TXNIP-siRNA present, and the glycolytic flux was detected in DMEM assay medium following sequential addition of glucose, oligomycin and 2-deoxy-glucose (2-DG, a hexokinase inhibitor). Consistent with the OCR finding, TXNIP deletion abolished the disturbed flow-mediated ECAR reduction that would otherwise be present under such conditions (Figure 5E). The quantification demonstrated that glycolysis and glycolytic capacity were significantly decreased in the disturbed flow group, compared to the laminar-flow group. Nevertheless, TXNIP depletion under disturbed flow conditions displayed similar bioenergetic profiles of glycolysis and glycolytic capacity as under laminar flow (Figure 5F). Thus, to better understand the specific physiological role of TXNIP in glucose metabolism, we selectively measured glucose metabolism-related gene expression in endothelial cells. We found that the glucose transporter type 4 (GLUT4), a major mediator of glucose removal from the circulation, was downregulated in disturbed flow group compared to the laminar flow group, while TXNIP deletion exhibited higher levels of this protein than the DF + NC-siRNA group (Figure 5G). This observed GLUT4 decrease was also associated with significantly lessened expression of PDH E1α (Figure 5G), which provides the primary link between glycolysis and the TCA cycle. Together, these data indicate that elevation of TXNIP expression blunted glucose uptake, suggesting that it is the key regulator mediating glucose utilization.

### TXNIP activation promoted the disturbed flow-induced pro-inflammatory response

**FIGURE 6:**
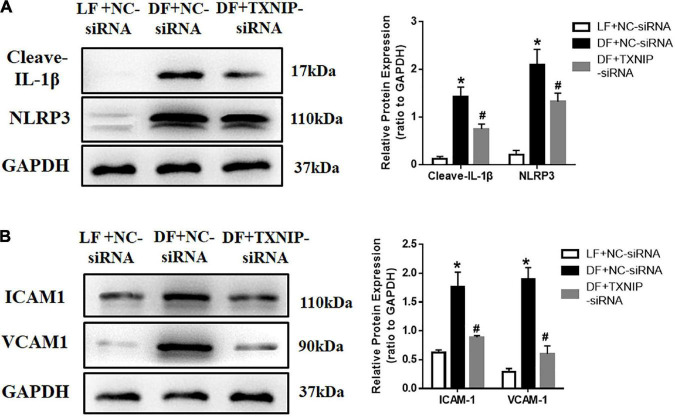
TXNIP activation promoted the disturbed flow-induced pro-inflammatory response. **(A)** Representative Western Blots, along with quantification of cleaved-IL-1β and NLRP3, **(B)** Representative Western Blots, along with VCAM1 and ICAM1 quantification, in the LF + NC-siRNA, DF + NC-siRNA, and DF + TXNIP-siRNA groups. The blot shows representative images of five independent experiments. Results are shown as mean ± SD. *P < 0.05 vs. LF + NC-siRNA, ^#^*P* < 0.05 vs. DF + NC-siRNA.

To investigate whether TXNIP mediated HUVEC pro-inflammatory response under different flow velocities, NLRP3 and cytokine interleukin (IL)-1β expression were examined by Western Blotting. The expression of the cleaved form of NLRP3 and IL-1β was upregulated in disturbed flow-treated HUVECs, but decreased upon TXNIP siRNA treatment (Figure 6A). Application of disturbed flow, but not laminar flow, upregulated the levels of cell adhesion molecules VCAM1 and ICAM1 in HUVECs. By contrast, such increases were offset by TXNIP siRNA treatment (Figure 6B). Taken together, these results indicated that shear stress induced HUVEC inflammation, in turn contributing to endothelial dysfunction.

## Discussion

**FIGURE 7:**
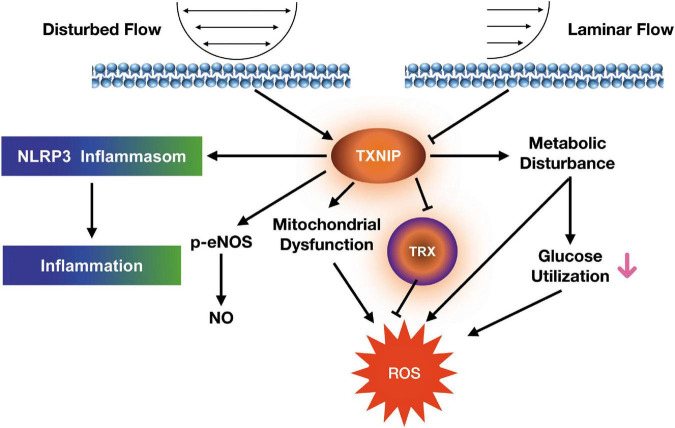
Proposed signaling mechanism linking shear stress to endothelial dysfunction in HUVECs.

TXNIP is a key skeletal muscle regulator of glucose usage and metabolism, as well as a recently found key inflammatory mediator (16–19). The plasma TXNIP levels were significantly increased in coronary of slow flow patients. Interestingly, patients with coronary slow flow do not exhibit slow flow and TXNIP enhancement in peripheral artery. The aim of our research was to investigate the effects of TXNIP on the relationships between oxidative metabolism and endothelial dysfunction in disturbed flow model. In light with the latter discovery, our findings demonstrated it serving a significant role in endothelial redox and inflammatory responses, where its ablation led to prominent reduction in cellular ROS and restoration of mitochondrial function. This reduction was found to be associated with lowered inflammatory response and NLRP3 expression. Furthermore, eliminating TXNIP also yields beneficial effects in the forms of increased glucose uptake, lactate and ECAR levels in a glucose-utilization study, as well as lowered HUVEC cell adhesion through its lowering of VCAM and ICAM expression. All these findings indicate TXNIP playing an important role in the development of HUVECs shear stress response and dysfunction. Figure 7 depicts our proposed mechanism for the dysfunctionality of endothelial cells under shear stress conditions.

Diabetic individuals, who also are more prone to atherosclerosis, demonstrate higher TXNIP expression levels. Previous studies showed that lower TXNIP expression enhanced skeletal muscle glucose uptake and improved glycaemic control (17), suggesting TXNIP inhibition being a potential diabetic intervention strategy. It was reported that endogenous NO can suppress TXNIP expression and that TXNIP facilitates nitrosative stress. However, the direct effects of TXNIP on NO regulation in disturbed flow-induced endothelial dysfunction were not investigated. In this study, TXNIP/NO interaction under shear forces was demonstrated *via* eNOS coupling regulation. In addition, our results indicated lower ATP levels, along with increased ROS and membrane potential depolarization in mitochondria from disturbed flow-treated cells, which were all reversed under TXNIP deletion, demonstrating the latter’s potential in preventing mitochondrial dysfunction. Overall, the results from this study have further clinical and therapeutic implications, where TXNIP ablation could be beneficial in diabetics *via* reducing vascular inflammation and dysfunction.

TXNIP expression has been previously demonstrated to be induced by a glucose-dependent signaling pathway (20). This connection is of significant physiological relevance, owing to the finding that after its induction, TXNIP negatively regulates glucose uptake (18, 21, 22). The operation of this regulatory pathway is as follows: Cells requiring more energy or “building blocks” for macromolecular synthesis demonstrate a higher glycolytic rate, and thus a decrease of levels for certain glycolytic metabolites. Both changes are sensed by the TXNIP transcriptional machinery to repress TXNIP expression. Based on our findings, the data suggest that ectopic TXNIP expression blunts glucose uptake and lactate production. Thus, we propose that TXNIP acts as a mediator to integrate cellular metabolic activity and energy requirements with cellular glucose supply, which may have important implications for endothelial cell glucose homeostasis regulation.

Several results suggest that TXNIP is a critical target for steady laminar flow-associated anti-inflammatory effects (7, 23). Our results show that steady laminar flow increased the ability of HUVECs to form capillary like structures, as well as lowering TXNIP expression and NLRP3-mediated inflammation. Thus, it is likely steady laminar flow inhibits TXNIP expression, leading to the limitation of HUVEC inflammation and endothelial function improvement. This study also investigated the potential role of TXNIP in disturbed flow-induced HUVEC inflammation response, where the results clearly demonstrated TXNIP ablation decreasing cellular ROS levels, as well as attenuating the stimulation of pro-inflammatory and pro-adhesion gene expression.

In summary, our data suggested TXNIP playing an important role in vascular inflammation, oxidative metabolism and mitochondrial dysfunction, with respect to endothelial dysfunction development. TXNIP expression modulation could therefore be a potential therapeutic strategy for intervention in flow velocity-related vascular complications.

## Data availability statement

The original contributions presented in this study are included in the article/supplementary material, further inquiries can be directed to the corresponding authors.

## Ethics statement

The studies involving human participants were reviewed and approved by the Medical and Health Ethics of Shenzhen People’s Hospital, Jinan University. The patients/participants provided their written informed consent to participate in this study.

## Author contributions

YW, JL, and XS performed the experiments and were major contributors in writing the manuscript. HL and RC performed the data analysis and interpretation, as well as being responsible for the study design, and manuscript drafting. JY, XYZ, and BL were responsible for statistical analysis. YW, SD, and XXZ prepared the reagents and revised the manuscript. ZX and JY designed the entire study and provided funding. All authors read and approved the final manuscript.
